# Dystrophin Expressing Chimeric (DEC) Human Cells Provide a Potential Therapy for Duchenne Muscular Dystrophy

**DOI:** 10.1007/s12015-018-9807-z

**Published:** 2018-03-15

**Authors:** Maria Siemionow, Joanna Cwykiel, Ahlke Heydemann, Jesus Garcia, Enza Marchese, Krzysztof Siemionow, Erzsebet Szilagyi

**Affiliations:** 1 Department of Surgery, Poznan University of Medical Sciences, Poznan, Poland; 2 Depatment of Orthopedics, University of Illinois at Chicago, Chicago, IL USA; 3 Department of Physiology and Biophysics, University of Illinois at Chicago, Chicago, IL USA; 4 Department of Clinical Health Sciences, Saint Louis University, Saint Louis, MO USA

**Keywords:** Duchenne muscular dystrophy, Stem cells, Myoblasts, Dystrophin expressing chimeric human cells, DEC therapy, *Mdx*/*scid* mice, Dystrophin, Ex vivo cell fusion, DMD, Transplant

## Abstract

Duchenne Muscular Dystrophy (DMD) is a progressive and lethal disease caused by mutations of the dystrophin gene. Currently no cure exists. Stem cell therapies targeting DMD are challenged by limited engraftment and rejection despite the use of immunosuppression. There is an urgent need to introduce new stem cell-based therapies that exhibit low allogenic profiles and improved cell engraftment. In this proof-of-concept study, we develop and test a new human stem cell-based approach to increase engraftment, limit rejection, and restore dystrophin expression in the *mdx*/*scid* mouse model of DMD. We introduce two Dystrophin Expressing Chimeric (DEC) cell lines created by ex vivo fusion of human myoblasts (MB) derived from two normal donors (MB^N1^/MB^N2^), and normal and DMD donors (MB^N^/MB^DMD^). The efficacy of fusion was confirmed by flow cytometry and confocal microscopy based on donor cell fluorescent labeling (PKH26/PKH67). In vitro, DEC displayed phenotype and genotype of donor parent cells, expressed dystrophin, and maintained proliferation and myogenic differentiation. In vivo, local delivery of both DEC lines (0.5 × 10^6^) restored dystrophin expression (17.27%±8.05—MB^N1^/MB^N2^ and 23.79%±3.82—MB^N^/MB^DMD^) which correlated with significant improvement of muscle force, contraction and tolerance to fatigue at 90 days after DEC transplant to the gastrocnemius muscles (GM) of dystrophin-deficient *mdx*/*scid* mice. This study establishes DEC as a potential therapy for DMD and other types of muscular dystrophies.

## Introduction

Duchenne Muscular Dystrophy (DMD) is a progressive and lethal disease, caused by X-linked mutations of the dystrophin encoding gene. The lack of dystrophin leads to weakness, degeneration, and consequent fibrosis in skeletal and cardiac muscles [1]. Currently, there is no cure for DMD patients. Preclinical and clinical approaches in the pipeline include exon skipping, gene editing via viral vectors, and stem cell transplants [2–4]. The recently developed gene splicing CRISPR system [5, 6] delivered by adeno-associated viruses demonstrated encouraging results in preclinical animal studies. However, clinical efficacy is still debatable due to safety concerns for off-target mutations and limitations of subsequent treatments due to sensitization [5, 7]. Allogeneic stem cell transplantation of satellite cells [8], mesenchymal stem cells [9], induced pluripotent stem cells [10], dermal fibroblast [11] and muscle derived stem cells [12] improved dystrophin expression in DMD small animal models with variable results. The limiting factor of successful stem cell engraftment is the allogenic immune response [13–15]. The use of immunosuppressive therapy supports the engraftment [16], however the efficacy is sub-optimal [17]. Thus, it is critical for the success of DMD stem cell-based therapies to exhibit low allogenic profiles, which will enhance and maintain engraftment.

Based on our experience with chimerism and tolerance induction in bone marrow and vascularized composite allotransplantation (VCA) [18–22] as well as encouraging results of our previously published proof-of-concept study which confirmed the feasibility of ex vivo cell fusion to create murine Dystrophin Expressing Chimeric (DEC) cells [23], we have developed and tested a new human stem cell-based line of chimeric cell therapy. This novel clinically relevant therapeutic approach has the potential to increase engraftment, limit rejection, and restore dystrophin expression in patients suffering from DMD and other types of muscular dystrophies (MD).

We introduce two Dystrophin Expressing Chimeric (DEC) human cell lines created by ex vivo fusion of human myoblasts (MB) derived from two normal donors (MB^N1^/MB^N2^), and normal and DMD donors (MB^N^/MB^DMD^).

In this study, we confirm feasibility of human DEC cell lines creation *via* ex vivo fusion using polyethylene glycol (PEG) technology. In vitro, DEC displayed phenotype and genotype of donor parent cells, expressed dystrophin, and maintained proliferation and myogenic differentiation. In vivo, DEC restored dystrophin expression which correlated with significantly improved muscle function at 90 days after intramuscular transplant to the *mdx*/*scid* mouse – the model of DMD. This study establishes DEC as a potential therapy for DMD, which addresses the limitations of current stem cell-based therapies.

## Materials and Methods

### Mice and Animal Care

This study was approved by the Institutional Animal Care and Use Committee (IACUC) of University of Illinois at Chicago, which is accredited by the American Association for the Accreditation of Laboratory Animal Care (AAALAC). All animals received humane care in compliance with the ‘Principles of Laboratory Animal Care’ formulated by the National Society for Medical Research and the ‘Guide for the Care and Use of Laboratory Animal Resources’ published by the US National Institutes of Health [24]. Six to eight-week old *mdx*/*scid* mice - animal model for Duchenne Muscular Dystrophy (B10ScSn.Cg-Prkdc^scid^ Dmd^*mdx*^/J, stock number 018018) with respective background wild type mice (C57BL/10ScSnJ, stock number 000476) were purchased from Jackson Laboratories for human DEC lines testing. Mice were housed in the Molecular Biology Research Building, an AAALAC-accredited animal facility, at University of Illinois at Chicago.

### Human Myoblast Culture

Cryopreserved normal human myoblasts were purchased from Lonza Inc (Mapleton, IL, USA), while DMD-affected myoblast were purchased from Axol Bioscience Ltd. (Little Chesterford, UK). Myoblasts (MB) were cultured in standard conditions in specific Skeletal Muscle Cell Growth Medium-2 (SkGM-2 bulletkit, Lonza Inc, Mapleton, IL, USA) supplemented with human Epidermal Growth Factor (hEGF), 0.5 ml; Fetuin, 5.0 ml; Bovine Serum Albumin (BSA), 5.0 ml; Dexamethasone, 0.5 ml; Insulin, 5.0 ml; Gentamicin/Amphotericin B (GA), 0.5 ml. Culture medium was changed twice a week and upon reaching 60–70% confluence, myoblasts were harvested and passaged using mechanical and enzymatic dissociation methods of 5 min incubation with 0.25% trypsin EDTA (Gibco-ThermoFischer, Waltham, MA USA). Enzymatic activity for cell detachment was stopped with culture media supplemented with 10% FBS (fetal bovine serum, Hyclone, GE Healthcare Bio-Sciences, Pittsburgh, PA, USA). Next, the cells were washed twice. Human MBs were harvested between passages 5–7, which is the optimal passage for ex vivo cell fusion procedure.

### Cell Fusion Procedure

After harvesting and counting in 0.4% Trypan Blue staining solution (Gibco- ThermoFischer, Waltham, MA, USA), parent myoblast (MB^N1^ and MB^N2^ or MB^N^ and MB^DMD^) were washed in serum-free DMEM culture media supplemented with antibiotics (1% Antibiotic–Antimycotic solution, Gibco- ThermoFischer, Waltham, MA, USA). Then, parent myoblasts (MB^N1^ and MB^N2^ or MB^N^ and MB^DMD^) were fluorescently labeled using either PKH26 or PKH67 (Sigma, St. Louis, MO, USA) tracking membrane dyes according to the manufacturer’s instructions. Briefly, each parent cell pellet was suspended in 1 ml diluent C (Sigma, St. Louis, MO, USA) and 4 µl PKH dye was added to the 2 ml total volume. After 4-min room temperature incubation, the staining procedure was stopped with the addition of 1% BSA and consequent wash in culture media. Before the fusion procedure, parent cells were mixed and washed in serum-free DMEM basal media. After the pellet was mobilized, the fusion procedure was performed using 1.46 g/mL PEG solution (PEG 4000, EMD) containing 16% DMSO (Sigma, St. Louis, MO, USA) [25]. The fused cells then were washed in complete culture media and transferred to PBS-based fluorescently activated cells sorting (FACS) buffer containing 5% HEPES, 1% EDTA and 5% FBS. Finally, cells presenting double (PKH26/PKH67) staining were selected via FACS (MoFlow Astrios, Beckman Coulter, San Jose, CA, USA) and used for further in vitro analysis or transplanted to recipient *mdx*/*scid* mice. A total of 6 cell fusions for each human DEC line (MB^N1^/MB^N2^ DEC and MB^N^/MB^DMD^ DEC) were performed for in vitro assays and 8 fusions for each DEC line were performed for in vivo DEC cell delivery.

### Flow Cytometry and Confocal Microscopy Analysis for Confirmation of DEC Fusion

Following fusion procedure, samples of sorted double stained PKH67/PKH26 labeled DEC, as well as corresponding single stained controls (PKH67 labeled MB^N1^ and MB^N^, and PKH26 labeled MB^N2^ and MB^DMD^) and unstained controls were fixed in 4% paraformaldehyde for 15 min. and washed in PBS. Samples for flow cytometry were analyzed using Fortessa (Beckman Coulter, San Jose, CA, USA). For confocal microscopy cells were spun onto positively charged lysine coated microscope slides and counterstained with DAPI (Vector mounting media with DAPI). Cells were examined on Zeiss Meta confocal microscope and images captured and analyzed with ZEN software (USA, St. Louis, MO, USA).

### PCR-STR and PCR-rSSOP DNA Profiling

DNA isolation was performed with DNeasy Blood and Tissue Isolation kit (Qiagen) according to manufacturer’s instructions. DNA samples of DEC and donor cells were typed using the polymerase chain reaction-reverse sequence-specific oligonucleotide probe (PCR-rSSOP) method using commercial kits (LABtype rSSO Typing Test, OLI) and polymerase chain reaction short-tandem repeat (PCR-STR). For the PCR-rSSOP, the sample DNA was subjected to PCR amplification (PE9700, Thermo cycler, Life technologies) in a 10 µL reaction volume, with the PCR run at 96 °C for 3 min, 96 °C for 20 s, 60 °C for 20 s, and 72 °C for 20 s, for 5 cycles, and 96 °C for 10 s, 60 °C for 15 s, and 72 °C for 20 s for 30 cycles followed by 72 °C for 10 min and stored at 4 °C. After amplification, the PCR products were denatured, and hybridized with the corresponding locus beads at 60 °C for 15 min, which were washed three times. Then, streptavidin-conjugated phycoerythrin (SAPE) was reacted with the products for 5 min at 60 °C, and following washing, the fluorescent products were detected using the Luminex 200 (Luminex, USA) and suspended in 60 µL wash buffer.

*For the PCR-STR*, 5 ng DNA was amplified using the ABI 3730*xl* DNA Analyzer with the Promega *GenPrint* 10 ® system (Promega, Madison, Wisconsin, USA). The 10 studied STR loci included: TH01, D21S11, D6S1043, D5S818, D13S317, D7S820, D16S539, CFS1PO, Amelogenin, vWA, TPOX. Amplification was conducted in a 25 µl reaction volume containing 5 µl of GenePrint Master Mix (Promega, Madison, Wisconsin, USA), 5 µL of GenePrint Primer Pair Mix (Promega, Madison, Wisconsin, USA), and DNA template. Appropriate negative and positive controls were used. The raw data were uploaded to GeneMapper® 5.0 analysis software (Applied Biosystems, Foster City, CA, USA) and allelic profile(s) were created according to analysis conditions supplied by Promega. The PCR conditions consisted of initial denaturation step at 96 °C for 5 min, followed by 30 cycles of 94 °C for 10 s, 59 °C for 1 min, and 72 °C for 30 s, with a final extension at 60 °C for 10 min and then a 4 °C incubation.

### Quantification of Dystrophin Expression by Taqman Real-Time PCR

#### RNA Isolation

Total RNA was isolated from tissues using TRIzol reagent (LifeTechnologies) per manufacturer’s instructions. Concentration and quality of extracted total RNA was measured spectrophotometrically with NanoDrop® ND-1000. The ratio of sample absorbance *A*_260/280_ < 1.8 was considered an acceptable measure of RNA purity.

Total RNA in concentration of 600 ng/µl was reverse-transcribed to cDNA in a total volume of 20 µl, using High Capacity cDNA Reverse Transcription Kit (Applied Biosystem) according to the manufacturer’s instructions. The amount of cDNA synthesized in a single reaction was sufficient to PCR-amplify all interrogated genes.

#### Relative Quantification of Dystrophin Expression by Real-Time PCR

Quantitative assessment of dystrophin expression (Hs00758098_m1) was performed using the 7300 Real-Time PCR detection system with 7300 System SDS software (Applied Biosystem). Amplification was carried out in a total volume of 25 µl containing TaqMan Universal PCR Master Mix (2x), and Gene Expression Assay Mix (20x). The reactions were cycled 40 times using the following parameters: 50 °C for 2 min, 95 °C for 10 min, 95 °C for 30 s and 60 °C for 1 min. All PCR runs were performed in triplicate to achieve reproducibility. Expression of all examined genes was compared to endogenous controls of GAPDH (Hs99999905_m1, Applied Biosystem).

### Immunofluorescence Detection of Dystrophin In vitro

MB^N1^/MB^N2^ DEC and MB^N^/MB^DMD^ DEC lines (n = 4 fusions/line) and parent myoblast lines (MB^N1^ and MB^N2^, MB^N^ and MB^DMD^, n = 4/cell type) were cultured in a Skeletal Muscle Cell Growth Medium-2 on poly-L-lysin coated German glass coverslips (Corning Inc, New York, USA) placed in 6-well plates (Corning, New York, USA). At 1, 7, 14 and 21 days of culturing, cells were fixed with ice-cold acetone for 10 min, washed, and unspecific antibody binding was blocked with 10% normal goat serum. Mouse monoclonal anti-human anti-dystrophin primary antibody (MANDYS8, 1:200, ThermoFischer, Waltham, MA, USA) and goat anti-mouse AlexaFluor-467 conjugated secondary antibody (1:400, ThermoFisher, Waltham, MA, USA) were used for dystrophin detection. Nuclei were counterstained with DAPI (Vector Laboratories, Burlingame, CA). A Zeiss Meta confocal microscope with ZEN software was used for fluorescence signal detection and analysis.

### Phenotype Analysis by Flow Cytometry

Myoblast phenotype markers were evaluated in both normal and DMD-affected parent myoblast populations (MB^N1^ and MB^N2^, MB^N^ and MB^DMD^ n = 4/line) as well as in fused DEC lines (MB^N1^/MB^N2^ DEC and MB^N^/MB^DMD^ DEC) 12 h after fusion (n = 4/line). The following antibodies were used: anti-human antibodies against CD34, CD90, CD45 and CD56 (BD Biosience, San Jose, CA, USA). Fluorescence detection was performed by flow cytometry (Beckman Coulter Gallios, San Jose, CA, USA) and results were analyzed by FlowJo software (FlowJo, LLC, Ashland, Oregon, USA).

### Proliferation Assay

Proliferation of parent cells (MB^N^ and MB^DMD^, n = 3/cell type) before cell fusion procedure and fused DEC lines (MB^N1^/MB^N2^ DEC and MB^N^/MB^DMD^ DEC; n = 3 fusions/line) was assessed by flow cytometry up to 21 day. Parent MB and DEC populations were prepared at a single-cell suspension to be labeled. Cells were washed two times with PBS to remove any serum. Cells were suspended in pre-warmed PBS. A 5 µM solution of cell proliferation dye eFluor™ 670 (eBioscience- ThermoFischer, Waltham, MA, USA) in PBS was used for labeling cells. After incubation for 10 min at 37 °C in the dark, labeling was stopped by adding 5 volumes of cold complete media (SKGM, containing 20% FBS) followed by incubation on ice for 5 min. Cells were then washed three times and cultured on 6-well plates for 1, 3, 6, 13, 17 and 21 days. Cells were harvested with a 5-min 0.25% EDTA-trypsin (Gibco- ThermoFischer, Waltham, MA, USA) incubation and fixed with 4% paraformaldehyde. Freshly stained and fixed cells were used as a negative (non-proliferating cells) control. Samples were analyzed by flow cytometery (Beckham Coulter Gallios, San Jose, CA). Detected fluorescence of eFluor™ 670 of each sample was presented as independent histograms. Fluorescence detected of PKH26 and PKH67 pre-fusion staining of DEC was measured as well, and shift of fluorescence mean values was correlated with the post-fusion eFluor™ 670 fluorescence.

### Myogenic Differentiation of DEC

To confirm myogenic differentiation potential of DEC, freshly fused DEC lines (MB^N1^/MB^N2^ DEC and MB^N^/MB^DMD^ DEC, n = 4 fusions/ line) and control normal myoblasts (MB^N^) were cultured on German glass coverslips (ThermoFischer, Waltham, MA, USA) in serum-free Myogenic Differentiation Media (PromoCell, USA) supplemented with 10 µg/ml insulin to induce myogenic differentiation for 7 days. To assess dystrophin and SMHC co-expression, cells were fixed with ice-cold acetone and unspecific antibody binding was blocked with 10% normal goat serum. Rabbit polyclonal anti-fast myosin skeletal heavy chain antibody (1:200, Abcam, Cambridge, MA, USA) and mouse monoclonal anti-dystrophin antibody (MANDYS8, 1:200, ThermoFischer, Waltham, MA, USA) were used for primary detection of dystrophin and myosin heavy chain. For α-sarcoglycan, β-sarcoglycan and desmin detection, cells were fixed with 4% paraformaldehyde and non-specific antibody binding blocked with 5% normal goat serum. Primary detection was obtained with the sarcoglycan complex antibodies rabbit anti α-sarcoglycan (1:50, DSHB Hybridoma Product IVD3(1)A9, University of Iowa, USA) and rabbit polyclonal anti-β-sarcoglycan (1:50, Novus Biologicals, Littleton, CO, USA). Rabbit polyclonal anti-desmin (1:100, Invitrogen, ThermoFischer, Waltham, MA, USA) was used for primary detection of desmin.

Goat anti-rabbit IgG Alexa Fluor 488 (1:500, Molecular Probes, ThermoFischer, Waltham, MA, USA) and goat anti-mouse IgG2a Alexa Fluor 647 (1:500, ThermoFisher, Waltham, MA, USA) fluorescently conjugated secondary antibodies were used for corresponding primary antibody visualization. Appropriate negative tissue controls and isotype controls were implemented in the experiments. Fluorescence images were captured on Zeiss Meta confocal microscope and fluorescence.

### Transplantation of DEC

DEC were counted and washed in sterile DPBS twice and transferred in 60µ l total volume PBS to tuberculin syringe with 27G needle (ThermoFischer, Waltham, MA, USA). Mice were anesthetized with 1.5% isoflurane inhalation and the skin on the left posterior calf was shaved and aseptically prepared. Based on a standard circle shaped template, six microinjections (10µ l/injection, total volume 60 µl) were delivered into the gastrocnemius muscle (GM). Animals were allowed to recover in a heated environment and promptly returned to the colony.

The following experimental groups were performed after randomization of age matched 6–8 weeks old *mdx*/*scid* recipients for follow-up of 7 and 90 days post-transplant: vehicle treatment (n = 12, 60µ l DPBS), not fused MB^N^ from each of the donors (n = 9, 0.25 × 10^6^ /donor- total 0.5 × 10^6^ in 60 µl DPBS), fused MB^N1^/MB^N2^ DEC (n = 9, 0.5 × 10^6^ in 60µ l DPBS) and fused MB^N^/MB^DMD^ DEC (n = 9, 0.5 × 10^6^ in 60µ l DPBS).

Animals’ follow-up consisted of in vivo muscle strength tests (grip strength and wire hanging) twice a week and on day 7 and day 90 in situ, and ex vivo muscle strength tests were performed.

### Histological and Immunofluorescence Analysis

Formalin fixed and paraffin embedded gastrocnemius muscles (GM) were cut at 5-micron sections. Sections were stained with hematoxylin-eosin to analyze muscle structure and to quantify the central nucleated regenerating fibers. Five standardized regions of three non-serial cross-sections of n = 6 animals/ group were analyzed and fibers with centrally positioned nuclei were counted and normalized to total nuclei number.

OCT embedded frozen GM muscle was cut with a cryotome (ThermoFisher, Waltham, MA, USA) at 4-micron section. Cross-sections were fixed with ice-cold acetone. Immunoblocking was performed with 10% normal goat serum in 1% BSA. Dystrophin expression was detected using a primary rabbit polyclonal anti-human anti-dystrophin antibody (ThermoFischer, Waltham, MA, USA) in combination with goat anti-rabbit Alexa Fluor (AF) 488 conjugated secondary antibody (ThermoFischer, Waltham, MA, USA). Nuclei were counterstained with DAPI (Vector). A Zeiss Meta confocal microscope with ZEN software (Carl Zeiss, Oberkochen, Germany) was used for fluorescence signal detection and analysis. The number of dystrophin-positive muscle fibers in five standardized regions of each cross-section were counted and normalized to total nuclei numbers; three, non-serial cross-sections were quantified in each animal (n = 3/group at day 7 and n = 6/group at day 90). The total number of dystrophin-positive fibers was normalized to the number of total nuclei. This quantification method was chosen over normalization to the total fiber count as fiber morphology and specifically the diameter varied over time between experimental groups due to the DMD pathology progression or potential therapeutic effect. The co-localization of HLA-ABC and dystrophin signal was detected using anti-human mouse anti-HLA class I (1:200, Abcam, Cambridge, MA,USA) in combination with goat anti-mouse AF467 conjugated secondary antibody (1:400, Abcam Cambridge, MA, USA). The co-localization of HLA-ABC and skeletal myosin heavy chain expression was detected using anti-human anti-HLA class I (1:200, Abcam, Cambridge, MA, USA) in combination with goat AF467 conjugated secondary antibody (1:400, Abcam Cambridge, MA, USA) and rabbit polyclonal anti-myosin heavy chain antibody (1:200, Abcam, Cambridge, MA, USA) as primary and goat anti-rabbit Alexa Fluor 647 (1:500, Molecular Probes, ThermoFischer, Waltham, MA, USA) conjugated secondary antibody. Nuclei were counterstained with DAPI. A Zeiss Meta confocal microscope with ZEN software (Carl Zeiss, Oberkochen, Germany) was used to detect fluorescence signal. Quantification of dystrophin positive revertant fibers was performed counting 50 adjacent fibers in 4 non-consecutive cross-sections of each animal (total 200 fibers) of a 0.05 mm^2^ surface. Results were expressed in percentages.

### Muscle Strength Evaluation

#### Wire Hanging and Grip Strength Test

Mice motor function was monitored up to 90-day endpoint; wire hanging test and modified grip strength test were performed twice a week on alternate days. The order of animal test performance was randomly assigned.

The wire hanging test was performed a maximum of three consecutive times to prevent animal training for negative performance and the wire hanging time was measured. Although this muscle force evaluation was not specific for the GM, it provided supportive information regarding the general muscle strength of the DEC injected vs. control animals. A modified grip strength test for posterior limbs [26, 27] was used to measure GM-specific force. Briefly, the hook of grip meter (Digital Force Gauge, HL-50) was placed to touch the mouse toes. Upon the presence of grip, the hook was pulled repeated 10 times and the average maximum peak was used for further analysis.

#### In Situ Muscle Force Test

In situ muscle force measurements were performed at 90-days endpoint (n = 4/group). In situ muscle force measurements were performed under isoflurane anesthesia. The Achilles tendon was dissected and tied with silk to a force transducer. The sciatic nerve was isolated and stimulated with a bipolar wire electrode. Muscle force was measured after optimal voltage and length were determined. Fatigue was measured after 10 min of submaximal tetanic stimulation as described previously [28]. The GM was kept moist during the whole procedure by continuous drip of Krebs–Henseleit solution (in mM: 130 NaCl, 5 KCl, 1 CaCl2, 1.1KH2PO4, 0.85 MgSO4, 0.6 MgCl2, 25 HEPES, 25 NaCO3, 11 glucose bubbled with 95% oxygen and 5% carbon dioxide). The impact of the drip did not introduce mechanical artifacts. Optimal passive tension was determined by stimulating the sciatic 6 s. The passive tension was increased every 3 twitches until the maximum force was recorded. Optimal voltage was re-determined after each test. When the optimal voltage changed, the data from the previous test were discarded and the test was repeated. To ensure proper voltage, a set of twitches was elicited beginning at 1.0 V with a 1-ms pulse every 3 s with gradual increases in voltage until maximum force was obtained. The voltage used for the experiments was 1.2 times the optimal voltage determined and was usually 2.0 V. After optimal voltage and length were determined, the nerve was stimulated every 3 s with 1-ms pulses for 10 repetitions. The amplitude of the twitches and the rates of force generation and relaxation were measured. Twitches were repeated throughout the test to verify that the optimal voltage and passive tension were maintained.

A 300-ms, 50-HZ burst of stimulation was applied to the nerve every 3 s for 10 min Fatigue is reported as the minimum force, usually at 10 min, as a fraction of the reference force. The reference force was recorded from the second contraction. In these measurements, a smaller number means greater fatigue. Potentiation was reported as the maximal force, usually within the first 40 s of the test, as a percent of reference force.

#### Ex Vivo Muscle Force Test

After euthanasia, the contractile and passive properties of the GM were measured ex vivo using the Aurora Scientific in vitro muscle test system [29]. After whole GM dissection including the Achilles tendon, GMs were placed in warmed (37 °C) Krebs–Henseleit solution in a Radnoti glass chamber tissue bath. The Achilles tendon and proximal pole of the muscles were attached to the force transducer with silk ties. Muscle force was measured after establishing optimal length through a standardized stimuli pattern until reaching maximal wave and maximal strain.

### Statistical Analysis

Data are expressed as mean ± SD. OriginPro 2017 software was used to perform statistical analysis. Student T-test and one-way ANOVA with Tukey post-hoc test for pairwise comparisons were used to define statistical significance. P values were considered significant below 0.05.

## Results

### Confirmation of Creation of Human DEC Lines *via* Ex vivo Fusion Procedure

**Fig. 1:**
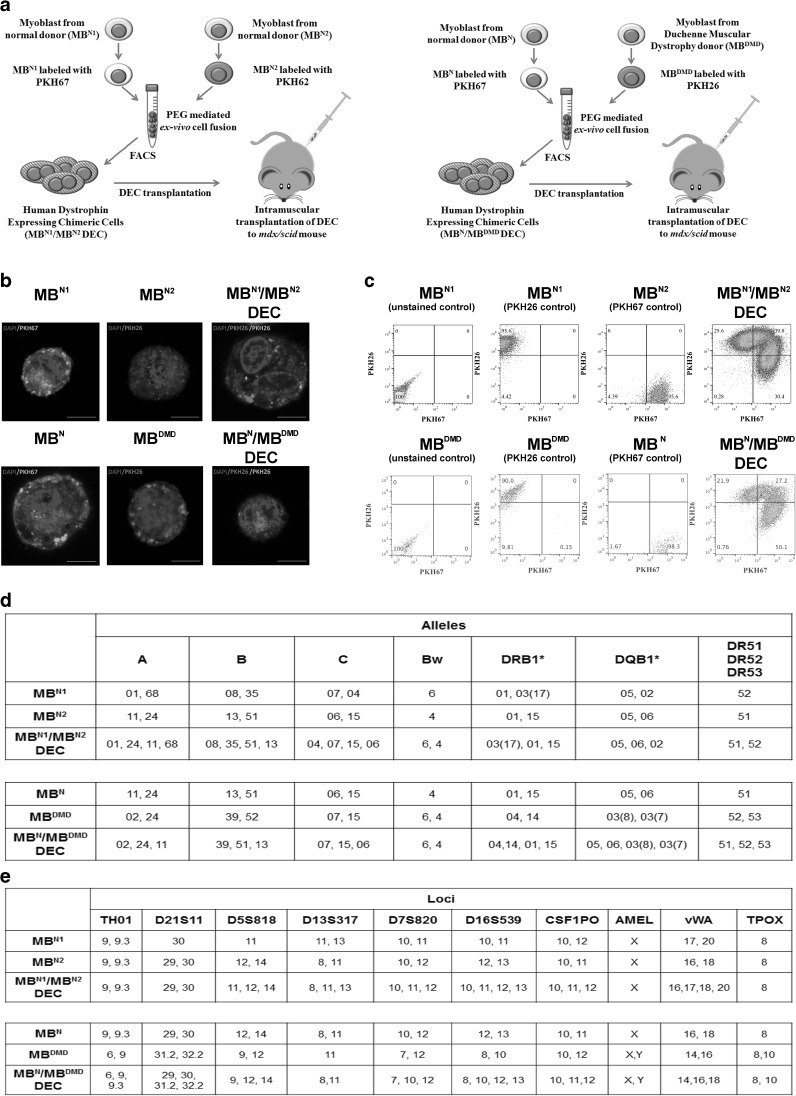
**Confirmation of ex vivo creation of two Dystrophin Expressing Chimeric (DEC) human cell lines derived from myoblasts of two normal donors (MB**^**N1**^/**MB**^**N2**^**) and from myoblasts of normal and DMD donors (MB**^**N**^/**MB**^**DMD**^**). a**) Diagram of ex vivo polyethylene glycol (PEG) mediated cell fusion procedure to create DEC; left panel, normal myoblast fusion (MB^N1^/MB^N2^), right panel, fusion of myoblast from normal and DMD-affected donor (MB^N^/MB^DMD^). (**b**) Representative immunofluorescence images of MB^N1^, MB^N^ (green), and MB^N2^, MB^DMD^ (red) parent cells before fusion, and MB^N1^/MB^N2^ DEC and MB^N^/MB^DMD^ DEC after fusion; For merge: Green: PKH67; Red: PKH26; Blue: DAPI (nuclei); Magnification 630X, scale bars 10 µm. (**c**) Fusion of the MB^N1^ and MB^N2^ parent cells (top) and MB^N^ and MB^DMD^ parent cells (bottom) assessed by FACS. The overlapping fluorescence of PKH67/PKH26 confirms chimeric state for MB^N1^/MB^N1^ DEC cell line (top - far right) and MB^N^/MB^DMD^ DEC cell line (bottom - far right). (**d**) PCR-rSSOP analysis of DEC for the presence of parent cell specific alleles from both donors. (**e**) STR-PCR analysis of DEC for the presence of parent cell specific loci from both donors

We developed, optimized and confirmed fusion procedure and created two novel human Dystrophin Expressing Chimeric (DEC) cell lines. First, from normal myoblast donors (MB^N1^/MB^N2^) as a proof-of-concept for ex vivo myoblast fusion and second, from normal and DMD-affected donor (MB^N^/ MB^DMD^) as a clinically relevant concept of fusion (Fig. 1a). To confirm cell fusion, parent cell populations were independently labeled with PKH26 and PKH67 cell membrane fluorescent dyes. Confocal microscopy confirmed a chimeric state of DEC by presence of overlapping fluorescence images (PKH26/PKH67) (Fig. 1b), and FACS sorting, which further proved heterologous cell fusion (Fig. 1c). Furthermore, analysis of human genotype of fused DEC by STR-PCR and rSSOP-PCR confirmed combination of loci and alleles specific for each parent cell of normal and DMD-affected myoblast donors (Fig. 1d, e).

### Confirmation of Myogenic Markers Expression in Normal and DMD-Affected Human Myoblasts (MB) Before Fusion and in Both DEC Lines After Fusion

**Fig. 2:**
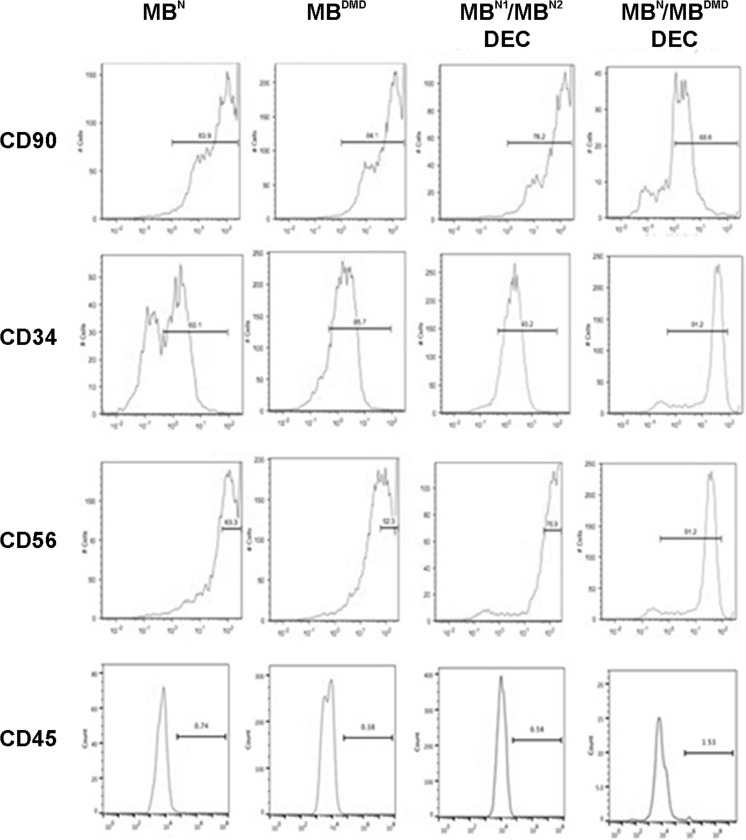
**Confirmation of myogenic phenotype in normal and DMD-affected human myoblasts (MB) before fusion and in the MB**^**N1**^/**MB**^**N2**^ **and MB**^**N**^/**MB**^**DMD**^ **DEC lines after fusion**. Representative flow cytometry plots of immunophenotyping for the expression of hematopoietic stem cell markers CD90, CD34, CD45 and myoblast-specific - CD56 marker of human myoblast (MB) from normal and DMD-affected donors before fusion and confirmation of maintenance of phenotype characteristics of MB^N1^/MB^N2^ and MB^N^/MB^DMD^ DEC lines after fusion

Phenotype analysis of normal (MB^N^) and DMD-affected (MB^DMD^) parent cells prior to and after ex vivo fusion, demonstrated continued expression of CD56, CD90, and CD34 markers indicating maintenance of myogenic lineage by both DEC lines after fusion (Fig. 2). Low expression (below 2%) of CD45, the marker of hematopoietic stem cells, combined with high expression of CD56 (85%), the myoblast specific marker, confirms high myogenic potential of DEC.

### In vitro Confirmation of Dystrophin Expression and Myogenic Differentiation of DEC Cell Lines After Fusion

**Fig. 3:**
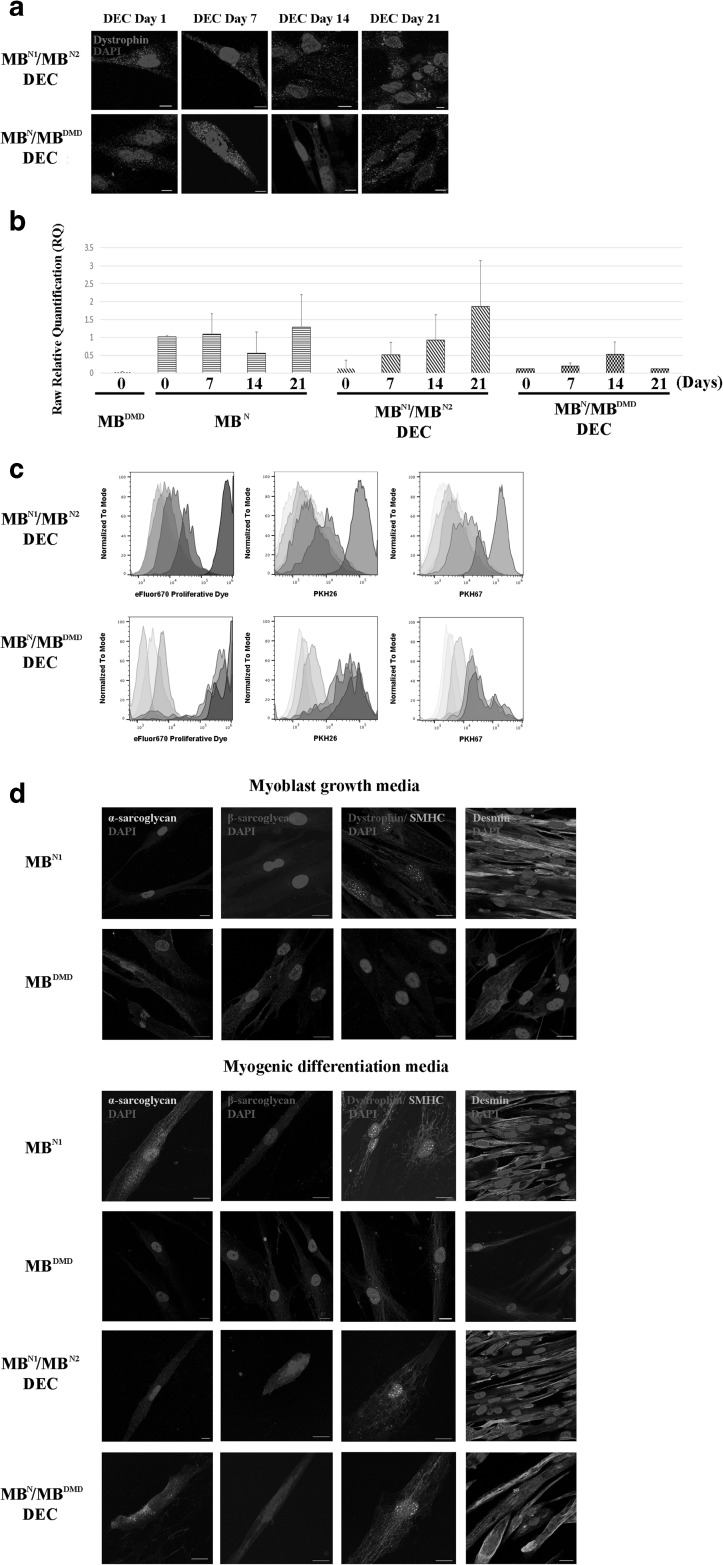
**Human DEC lines of both, normal (MB**^**N1**^/**MB**^**N2**^**) and normal and DMD donor (MB**^**N**^/**MB**^**DMD**^**) origin, maintain dystrophin expression, proliferation properties and undergo myogenic differentiation in culture**. (**a**) Representative immunofluorescence images of dystrophin expression (magenta) in MB^N1^/MB^N2^ and MB^N^/MB^DMD^ DEC in vitro up to 21 days after fusion (n = 4, magnification 400X, scale bar 10 µm). (**b**) Dystrophin expression in cultured MB^N1^/MB^N2^ and MB^N^/MB^DMD^ DEC up to 21 days after fusion quantified by Taqman PCR (n = 3, mean ± SD). (**c**) Flow cytometry analysis confirming proliferation of MB^N1^/MB^N2^ (upper row) and MB^N^/MB^DMD^ (lower row) DEC up to day 21 post-fusion. Decrease in the intensity of eFluor 670 Proliferative Dye correlated with decrease in the intensity of double PKH67/PKH26 staining used to confirm DEC fusion. (**d**) Immunofluorescence images of MB^N1^/MB^N2^ and MB^N^/MB^DMD^ DEC expressing sarcolemmal glycoproteins, alpha-sarcoglycan (yellow), beta-sarcoglycan (violet) and dystrophin (red) co-expressed with the motor protein skeletal myosin heavy chain (SMHC, green), and fusion protein desmin (green) after 7 days of stimulation in the myogenic differentiation media. Upper panel: Immunofluorescence images of normal (MB^N^) and DMD-affected (MB^DMD^) undifferentiated myoblast controls before fusion confirming lack of dystrophin expression in MB^DMD^ and a diffused distribution of SMHC in both MB^N^ and MB^DMD^. Lower panel: Immunofluorescence images of MB^N^, MB^DMD^, MB^N1^/MB^N2^ and MB^N^/MB^DMD^ DEC after 7-day stimulation in the myogenic differentiation medium confirming expression of dystrophin–glycoprotein complex (DGC) in MB^N^/MB^DMD^ DEC after fusion suggesting restoration of functional DGC. Uniform distribution of alpha-sarcoglycan and beta-sarcoglycan was observed after 7 days of induced differentiation further confirming progression of myogenic maturation. Nuclei were counterstained with DAPI (blue). Scale bar 20 µm

To evaluate therapeutic potential of DEC in vitro, dystrophin expression was detected by IF in both DEC cell lines up to 21 days after fusion (Fig. 3a). Quantitative analysis by real-time PCR (Fig. 3b) further confirmed an increase in dystrophin expression in DEC lines under in vitro culture conditions.

### Confirmation of DEC Proliferative Potential After Fusion

We confirmed proliferative potential of fused DEC in culture by eFluor670 proliferation dye dilution assay. Progressive decrease of eFluor670 intensity in DEC, correlated with decrease of fluorescent intensity of cell membrane dyes used for parent cell labeling prior to cell fusion (Fig. 3c). This indicates maintenance of proliferative potential of DEC lines after fusion assuring DEC survival and efficacy of PEG fusion technology.

### Confirmation of Differentiation Potential and Restoration of Functional Dystrophin- Glycoprotein Complex (DGC) After DEC Fusion

To test differentiation potential of DEC lines after fusion, we placed cells in myogenic differentiation medium and after 7 days observed co-expression of skeletal myosin heavy chain (SMHC) and dystrophin indicative of maintenance of myogenic differentiation potential of DEC. Moreover, we confirmed presence of desmin and proteins of the dystrophin–glycoprotein complex (DGC) in DEC after fusion of normal and DMD-affected myoblasts suggesting restoration of a functional DGC (Fig. 3d).

### Confirmation of DEC Engraftment and Dystrophin Expression In vivo at 90 Days After DEC Transplant to the GM of *mdx*/*scid* Mice

**Fig. 4:**
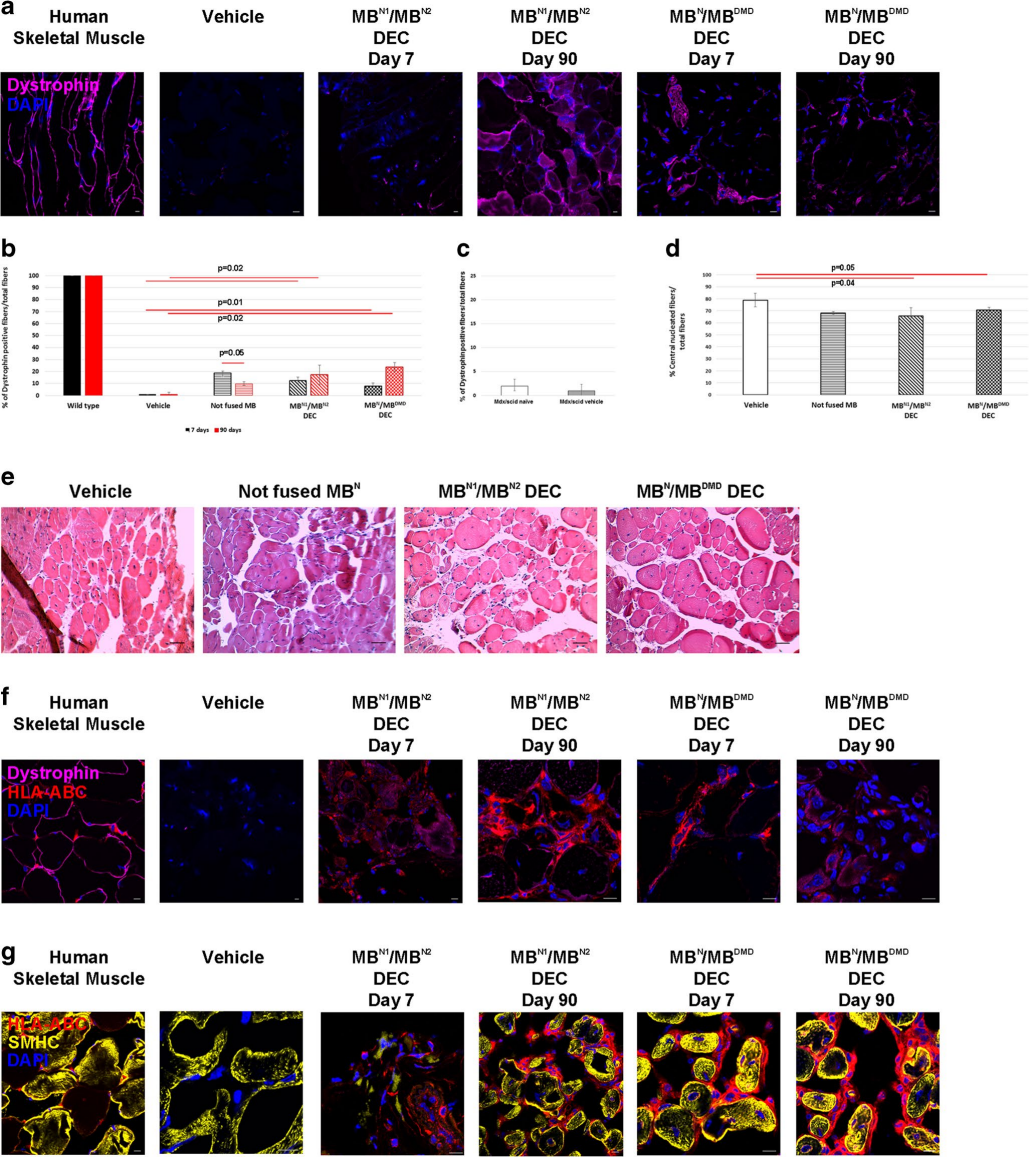
**Both DEC lines (MB**^**N1**^/**MB**^**N2**^ **and MB**^**N**^/**MB**^**DMD**^**) engraft, differentiate into skeletal muscles and maintain dystrophin expression up to 90 days after intramuscular transplantation to the gastrocnemius muscle (GM) of the** ***mdx***/***scid*** **mouse**. (**a**) Representative immunofluorescence images presenting restoration of dystrophin expression (magenta) in the GM of *mdx*/*scid* mice injected with MB^N1^/MB^N2^ and MB^N^/MB^DMD^ DEC. (**b**) Quantification of dystrophin expressing muscle fibers for both DEC lines: MB^N1^/MB^N2^ (12.3%± 2.5 at day 7 and 17.27%± 8.05 at day 90, n = 6) and MB^N^/ MB^DMD^ (7.42%± 3.25 at day 7 and 23.79%±3.82 at day 90, n = 6) after DEC transplant to the GM of *mdx*/*scid* mice (mean ± SD, p < 0.05 *vs*. vehicle). Dystrophin positive fiber counts were normalized to the total nuclei count within the region of interest (12ROI/sample). (**c**) Quantification of the dystrophin positive myocytes in GM of naïve and vehicle injected *mdx*/*scid* mice confirming low number (2%) of revertant fibers. Dystrophin expression in GM of age-matched naïve (n = 3) and vehicle-injected (n = 4) *mdx*/*scid* mice (mean ± SDM). (**d**) Quantification of the number of central nucleated muscle fibers in the GM injected with DEC indicates improvement in the dystrophic muscle (mean ± SD, p ≤ 0.05). (**e**) Representative images of hematoxylin & eosin (H&E) stained GM cross-sections of *mdx*/*scid* at 90 days after injection with vehicle, not fused MB^N^, MB^N1^/MB^N2^ and MB^N^/MB^DMD^ DEC, quantified for number of centrally nucleated muscle fibers. Images of five regions of interest (ROI) were used in three non-serial sections of each treated GM harvested from six animals/group (scale bar 50µ m). GM injected with DEC lines (MB^N1^/MB^N2^ DEC, MB^N^/MB^DMD^ DEC) and not-fused MB^N^ showed reduced number of muscle fibers with centrally located nuclei when compared to vehicle treated animals (controls), indicating restoration towards mature muscle fibers. (n = 6, mean ± SD, p < 0.05, One –way ANOVA). (**f**) Representative fluorescence images of dystrophin (green) expression co-localized with the expression of HLA-ABC (red) detecting human origin of the dystrophin positive muscle fibers (magnification 400X, scale bar 10µ m). (**g**) Representative fluorescence images of differentiated myofibers of DEC origin in the GM of *mdx*/*scid* mice detected by co-localization of human specific skeletal myosin heavy chain (SMHC, yellow) and HLA-ABC staining (red); (Magnification 400X, scale bar 10µ m)

We confirmed efficacy of engraftment and dystrophin restoration in vivo after transplantation of MB^N1^/MB^N2^ and MB^N^/ MB^DMD^ chimeric DEC lines (0.5 × 10^6^) to the GM of *mdx*/*scid* mice. IF confirmed early (7 days) and long-term (90 days) engraftment and dystrophin expression in MB^N1^/MB^N2^ and MB^N^/MB^DMD^ injected muscles compared to vehicle-injected control *mdx*/*scid* mice (Fig. 4a). Quantification of dystrophin expressing fibers at 7 and 90 days after DEC transplant confirmed significant increase in both MB^N1^/MB^N2^ (12.3%± 2.5 and 17.27%± 8.05, respectively) and MB^N^/ MB^DMD^ (7.42%± 3.25 at 7 days and 23.79%±3.82 at 90 days) injected muscles as compared to vehicle-injected controls. Muscles transplanted with non-fused normal myoblasts revealed dystrophin positive fibers at day 7 (18.81%± 3.06), which significantly declined by day 90 (9.89%± 1.51, p = 0.05; Fig. 4b). In order to exclude the possible role of spontaneously occurring conversion of *mdx*/*scid* muscle fibers towards revertant dystrophin expressing fibers, the revertant fibers were quantified based on the dystrophin expression in GM samples of naïve and vehicle-injected *mdx*/*scid* mice and revealed to be no more than 2% when normalized to the total nuclei counts (Fig. 4c). Structural analysis of hematoxylin & eosin (H&E) stained cross-sections of GM injected with DEC lines revealed decreased numbers of centrally nucleated fibers in DEC- injected muscles compared to vehicle treated animals (controls), indicating restoration towards mature muscle (Fig. 4d, e).

Restoration of dystrophin expression after DEC transplantation was confirmed by IF and human origin of dystrophin was confirmed by co-localization with HLA-ABC in the GM of *mdx*/*scid* mice injected with both DEC lines (Fig. 4f).

Human origin of the differentiated myofibers at 90 days after muscle injection with DEC cells was confirmed by co-expression of HLA-ABC, a human cell surface marker, and skeletal myosin heavy chain (SMHC), marker of myoblast differentiation (Fig. 4g).

### Assessment of Functional Outcomes 90 Days After DEC Transplant to the GM of *mdx*/*scid* Mice

**Fig. 5:**
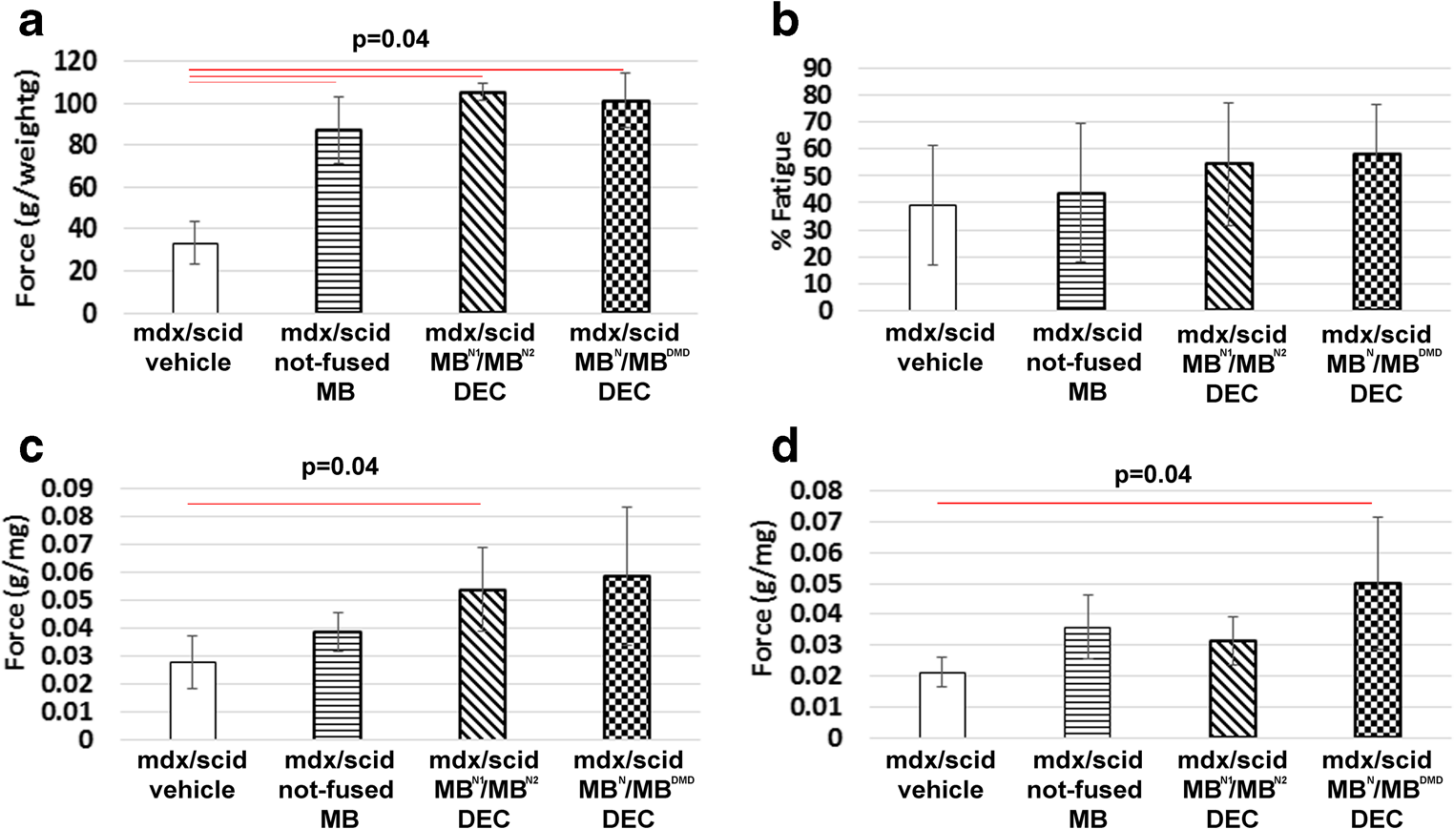
**DEC lines (MB**^**N1**^/**MB**^**N2**^**and MB**^**N**^/**MB**^**DMD**^**) improve muscle force 90 days after intramuscular transplant to GM of** ***mdx***/***scid*** **mice**. In situ muscle force (**a**) fatigue tolerance (**b**) and ex vivo muscle force under maximal sine wave stimulation (**c**) and maximal percent strain (**d**) in GM of vehicle, not-fused MB, and chimeric cells MB^N1^/MB^N2^ and MB^N^/MB^DMD^ injected *mdx*/*scid* mice. Muscle force was normalized by the isolated GM weight. (**a, b**; n = 4) (**c, d**; n = 3) p = 0.04, One-way Anova with Tukey post-hoc test

**Fig. 6:**
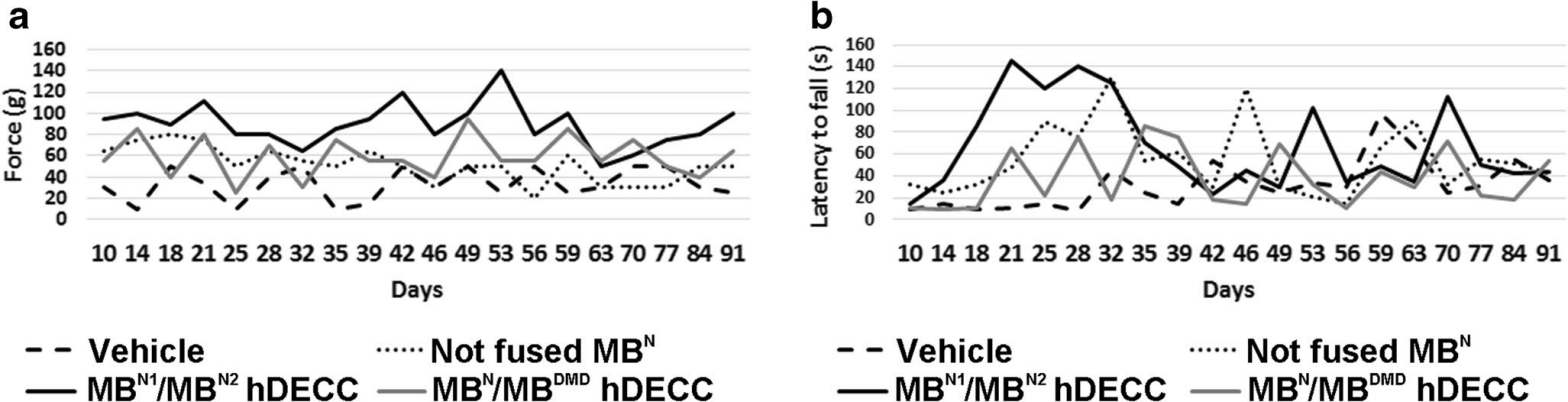
**Improvement of grip strength and wire hanging time observed up to 90 days after MB**^**N1**^/**MB**^**N2**^ and **MB**^**N**^/**MB**^**DMD**^ **DEC transplant to GM of** ***mdx***/***scid*** **mice**. Measurements of (**a**) grip strength expressed in mean muscle force (g) and (**b**) wire hanging time expressed in latency to fall (s) after injection of vehicle, not-fused MB^N^ and fused MB^N1^/MB^N2^ and MB^N^/MB^DMD^ DEC in GM of *mdx*/*scid* mice. During 90-day follow-up period, trends of increased muscle strength were observed in mice injected with both DEC lines; for MB^N1^/MB^N2^ between 42 and 59 days and for MB^N^/MB^DMD^ between 49 and 70 days, compared to vehicle controls and not fused MB^N^ injected *mdx*/*scid* mice. Improved trends in latencies to fall off the wire were observed between 18 and 28 days for MB^N1^/MB^N2^ and between 35 and 39 days for MB^N^/MB^DMD^ DEC injected mice compared to the control vehicle and not fused MB^N^ injected host. Behavioral processes and learning of experimental mice resulted in increased standard deviation for grip strength and wire hanging time assessment, limiting statistical power and consistency of these measurements

To evaluate the post-transplant efficacy of DEC on GM function, we subjected *mdx*/*scid* mice injected intramuscularly with vehicle and chimeric cell lines (MB^N1^/MB^N2^ and MB^N^/MB^DMD^) to in situ muscle force assessments and ex vivo muscle force measurements at 90 days post-transplant (Fig. 5). MB^N1^/MB^N2^ and MB^N^/MB^DMD^ injected *mdx*/*scid* mice had significantly improved in vivo muscle force (Fig. 5a) and tolerance to fatigue after 10-min tetanic stimulation (Fig. 5b). In addition, ex vivo muscle force measurement showed improved muscle contractions under maximal stimulation and strain in muscles injected with DEC compared to vehicle –injected controls (Fig. 5c, d). These findings were in line with the results of grip strength and wire hanging tests, which showed an improved trend in muscle function in DEC (Fig. 6) injected mice; however, due to the behavioral /cognitive learning bias influencing test outcomes, we used these results as supportive data.

## Discussion

Duchenne Muscular Dystrophy (DMD) is the most common and severe form of muscular dystrophies (MD) primarily affecting boys. It is caused by X-linked mutations in the dystrophin encoding genes. Currently, no cure exists for DMD patients. Different options to treat DMD have been tested, including gene therapies and CRISPR/Cas9 [7]. Despite promising pre-clinical results there are still concerns with the off-target mutations, limited applicability and sensitization. Thus, stem cell-based therapies have been widely investigated as alternative therapy for MD. Mesenchymal stem cells (MSC) are good candidates for MD treatment as they proliferate rapidly, undergo myogenic conversion and exhibit immunomodulatory properties. Unfortunately, their limited survival and myogenic differentiation in defected muscles remains a problem. Satellite cells are considered as the best cell population due to their capacity to generate new muscle fibers after transplantation to damaged muscles. However, their clinical application is challenged by the limitation of cell propagation resulting in low cell number and low rate of engraftment. Thus, myoblasts have been considered for cell-based DMD therapy and were applied in number of experimental and clinical studies [29]. Despite promising results, the clinical efficacy of myoblast therapies is limited due to the need of immunosuppression to support engraftment and lack of long-term sustainability after transplant [4, 30].

In the search of new stem cell-based therapies for tolerance induction in Vascularized Composite Allotransplantation, (VCA), we created bone marrow derived donor-recipient chimeric cells (DRCC) in vivo via adoptive transfer and ex vivo via PEG fusion [19, 21, 22]. DRCC therapy induced stable donor-derived mixed chimerism, mitigated immune recognition and allogeneic response, ultimately leading to long-term allograft survival. The previously reported reproducible, long-term engraftment and tolerogenic properties of chimeric cells were appealing as a potential DMD therapy.

Hence, we applied the concept of cell fusion technology to create human chimeric cell lines of myoblast origin as a novel, dystrophin delivery platform, presenting the allogeneic donor in the context of “self” in order to minimize the immune response and eliminate the need for immunosuppression. Previously, we have tested engraftment and dystrophin expression of murine MB^wt^/MB^*mdx*^ chimeric cells at 30 days after intramuscular transplant into GM of *mdx* recipients. Significant increase in dystrophin expressing fibers was confirmed in chimeric cell injected muscles compared to vehicle injected control mice (37.27% *vs*. 0.5%, respectively) [23]. In the current study, we created two new lines of Dystrophin Expressing Chimeric (DEC) human cells through ex vivo PEG fusion of normal and dystrophin-deficient myoblasts. Immunophenotype analysis of normal (MB^N^) and DMD affected (MB^DMD^) parent cells prior to cell fusion compared with the fused DEC lines (MB^N1^/MB^N2^ DEC and MB^N^/MB^DMD^) reveled expression of stem cell markers for CD90, CD34, and myoblast-specific marker -CD56 confirming maintenance of the myogenic phenotype after ex vivo fusion procedure. Furthermore, low expression (below 2%) of CD45, the hematopoietic stem cell marker, combined with high expression of CD56 (85%), the myoblast specific marker, confirms high myogenic potential of DEC. The efficacy of DEC therapy is cell-passage dependent and the optimal MBs passage for ex vivo fusion ranges between passage 5 and 7, since at passage 4 cells do not reach the required number and after passage 7 differentiation potential of MBs decreases [31, 32]. The genotype stability of both DEC lines was preserved after fusion as confirmed by STR-PCR and PCR-rSSOP profiling. The maintenance of differentiation potential and restoration of DGC was proven in vitro for both DEC cell lines after fusion. Moreover, the lack of DGC expression in myoblasts from DMD donors (MB^DMD^) before fusion as well as presence and maintenance of DGC expression in the created DEC line (MB^N^/MB^DMD^) after fusion suggests the potential of DEC technology as an effective tool for dystrophin delivery in DMD. Characterization of DEC in vitro up to 21 days after fusion demonstrated expression of dystrophin with a pattern of dystrophin distribution similar to what has been previously reported [33, 34] and comparable to normal myoblasts. Interestingly, we found that muscles injected with non-fused myoblasts showed decrease in the number of dystrophin positive fibers at day 90 when compared to day 7 post-transplant, confirming limited survival of non-fused myoblasts. Since high number of fibers with centrally positioned nuclei is a hallmark of degeneration/regeneration processes in DMD [35], our findings suggest that restoration of dystrophin in muscle fibers by differentiating DEC after transplant may stabilize dystrophic muscle environment and inhibit degeneration.

After DEC transplantation, long-term engraftment correlated with restoration of dystrophin expression and significant functional improvement of the gastrocnemius muscles of *mdx*/*scid* mice up to 90 days post-transplant. These findings were also reproduced in the *mdx* mouse model after murine DEC injection [23]. We demonstrated that the ex vivo fusion is a feasible and effective technology to deliver dystrophin to the dystrophin-deficient *mdx*/*scid* mouse host. In addition, DEC creation does not require cellular reprogramming, genome-editing, viral vector induced engineering, and as such has the potential to be safer and closer to clinical application. DEC therapy showed no short-term post-transplantation adverse events and no tumor formation during the follow-up observation and autopsy.

Our approach combines features of clinically established myoblast based therapies, where normal myoblasts with functional dystrophin derived from a closely related donor (e.g. father) are transplanted to the DMD-affected son under immunosuppression protocol [36]. To enhance engraftment and eliminate immunosuppression, DEC therapy represents a novel concept of ex-vivo fusion of normal (e.g. father) and DMD- affected (son) myoblasts and presents the allogenic/father’s myoblasts in the context of “self” (son), thus, minimizing immune response and the need for immunosuppression. The DEC treatment can be repeated over time and this provides the advantage over gene therapies or viral vector based therapies for DMD.

The successful long-term engraftment, maintenance of proliferative and myogenic properties and enhancement of function, qualifies DEC as a novel stem cell-based personalized therapy for restoration of muscle function in DMD patients, alone or in combination with other therapies where dosing is limited or sensitization occurs.
